# Verbal memory and executive components of recall in adolescent binge drinkers

**DOI:** 10.3389/fpsyg.2023.1239716

**Published:** 2023-10-23

**Authors:** Claudia Porras Truque, Luis Miguel García Moreno, Patricia Mateos Gordo, Xavier G. Ordoñez, Fernando Cadaveira, Montserrat Corral

**Affiliations:** ^1^Department of Psychobiology and Methodology in Behavioral Sciences, Universidad Complutense de Madrid (UCM), Madrid, Spain; ^2^Department of Research and Psychology in Education, Universidad Complutense de Madrid (UCM), Madrid, Spain; ^3^Department of Clinical Psychology and Psychobiology, Universidad de Santiago de Compostela (USC), Santiago de Compostela, Spain

**Keywords:** adolescence, alcohol, binge drinking, executive functioning, verbal memory

## Abstract

**Introduction:**

Binge drinking (BD) is a common health-risk behavior among young people. Due to the incomplete maturation of the adolescent brain, BD can lead to structural and functional changes that impact neurocognitive processes, particularly executive functioning and verbal memory. This study aimed to investigate the influence of executive components, such as mnemonic strategies and error avoidance, on performance in a verbal memory test and the potential effects of BD on this performance.

**Methods:**

A sample of 160 college students (51.55% female) with a mean age of 18.12 ± 0.32 years completed assessments for alcohol use disorders using the Alcohol Use Disorder Identification Test (AUDIT), as well as psychopathological (Symptom Checklist-90-R) and neuropsychological evaluations (Verbal Learning Test Spain-Complutense and WMS-III Logical Memory). The Intensive Drinking Evaluation Instrument (IECI) was utilized to gather detailed information about binge drinking habits, including the calculation of the highest blood alcohol concentration (BAC) during an episode of intake.

**Results:**

Correlation and clustering analyses revealed a negative association between BAC values and verbal memory performance, as well as the use of memory strategies. The high BAC group (BD) exhibited negative values in verbal memory variables, higher accuracy errors, and less efficient strategy usage, while the low BAC group (No BD) demonstrated better memory test performance, fewer precision errors, and superior use of memory strategies.

**Discussion:**

These findings support the hypothesis that, when solving tests requiring verbal memory, adolescents reporting a BD consumption pattern show fewer executive skills in their resolution and, therefore, achieved poorer performance than non-binge drinkers. Addressing excessive alcohol consumption in young individuals is crucial for safeguarding their cognitive development and overall well-being.

## 1. Introduction

Adolescence is a critical developmental phase. The adolescent brain undergoes structural and functional changes essential for optimal cognitive, behavioral, and emotional maturation throughout this stage (Konrad et al., 2013; Fuhrmann et al., 2015; Spear, 2018). Some characteristics of adolescence such as heightened reward sensitivity, experience seeking, impulsivity and poor self-control predispose them to engage in risky behavior, with alcohol use being the most frequent (Lees et al., 2020; Andrews et al., 2021). According to the World Health Organization (WHO), most adolescents start consuming alcohol between the ages of 12 and 16. Specifically, in Europe, 25% of adolescents start consuming alcohol at the age of 13 and the prevalence of weekly alcohol consumption among adolescents, although varying by region and gender, is between 2 and 33% (World Health Organization, 2019).

A common form of alcohol consumption among young people is called Binge Drinking (BD), which is characterized by the discontinuous consumption of quantities of alcohol that raises blood alcohol concentrations (BAC) up to 0.08 g/dL (Substance Abuse and Mental Health Administration, 2018; National Institute on Alcohol Abuse and Alcoholism, 2021). Many studies have demonstrated the detrimental effect of BD on different cognitive functions during adolescence (Nguyen-Louie et al., 2016; Carbia et al., 2017; Jones et al., 2017; Mahedy et al., 2018; Gierski et al., 2020). According to the meta-analysis by Lees et al. (2019), BD has structural and functional neural consequences among adolescents and young adults in the brain systems associated with reward systems and executive control. Neuropsychologically, several studies report specific cognitive deficits in verbal memory (Nguyen-Louie et al., 2016), working memory (Mahedy et al., 2018), visuospatial functioning (Winward et al., 2014), as well as decrements in attention, learning and executive functions (Gil-Hernandez and Garcia-Moreno, 2016; Carbia et al., 2018; Lees et al., 2019).

The term executive functions is used in reference to a group of complex top-down control processes that encompass working memory, inhibition, cognitive flexibility, and planning. These processes regulate non-automatic process and depend on the integrity of frontal circuits (Diamond, 2013). The developmental peak of the prefrontal regions of the brain occurs during adolescence and early adulthood. As a result, executive functions continue to improve throughout this stage (Miller and Cohen, 2001; Dumontheil, 2016; Berthelsen et al., 2017). According to the systematic review by Carbia et al. (2018), BD during adolescence and early adulthood is consistently associated with detriment to inhibitory control and cognitive flexibility. Furthermore, they suggest that BD seems to affect the more demanding working memory tasks that require information monitoring and executive strategies to compensate for a deficit in storage capacity. Other studies have not found any detrimental performance in typical neuropsychological assessment tests measuring executive functioning in adolescents with a short history of alcohol consumption, but it was observed in the performance of daily activities (Gil-Hernandez and Garcia-Moreno, 2016). They suggest that BD does not have an obvious neuro-cognitive impact in early stages of alcohol consumption, or that the adolescent brain can develop compensatory strategies.

Memory is one of the cognitive processes most affected by alcohol consumption, as the medial temporal regions on which it depends (DeMaster et al., 2014; Kitamura et al., 2017) are particularly vulnerable to its effects (Abrahao et al., 2017; Hermens and Lagopoulos, 2018). The prefrontal cortex, which supports executive functions, is also involved in memory functioning (Eichenbaum, 2017). Indeed, the efficiency of connectivity between the prefrontal and temporomedial circuits is very important in memory performance (Riggins et al., 2016; Guise and Shapiro, 2017), and these connections are dysfunctional in people with alcohol-related memory deficit (Nunes et al., 2019). The influence of executive functions on memory is primarily attributed to their regulatory role in propositional behavior processing rather than the direct processing of stimulus content (Miller and Cohen, 2001). This guiding action consists of organizing, classifying information, as well as choosing, designing plans, regulating, and evaluating behavior, giving flexibility to the proposed task (Tse et al., 2011; Milivojevic et al., 2015). The executive contribution can be considered as the enhancement of learning and memory efficiency through the utilization of cognitive strategies, particularly within the framework of metacognitive and meta-memory processes. Indeed, BD subjects with lower executive performance manifest inadequate use of semantic-type strategies and impaired learning interference, resulting in poorer performance in verbal memory tests (Carbia et al., 2018; Gierski et al., 2020). Recent studies suggest that BD subjects experience difficulties in executive functions and verbal memory, which are manifested as poor performance in tasks involving the recall of word lists guided by the creation of semantic groupings (Carbia et al., 2018; Kang and Kim, 2022; Rodríguez Holguín et al., 2023).

Learning word lists is one of the most common paradigms in neuropsychological verbal memory research (Flores-Lázaro et al., 2017; Lau et al., 2018; Pflueger et al., 2018; McAndrews et al., 2020). Literature describes adolescence as the most intense period for declarative memory development, which is mediated by information monitoring and cognitive control (Flores-Lázaro et al., 2017; Johnson et al., 2018). This control relies on the action of the executive components on memory content (Fernandez-Duque et al., 2000; Shimamura, 2000), as, for example, the use of grouping strategies (Roebers et al., 2007; Ghetti and Angelini, 2008).

Semantic clustering is the strategy most related to better memory performance, while the serial strategy does so in a less demanding way (Introzzi et al., 2010). Semantic clustering is a high-level cognitive process for encoding new information that involves deep processing and has been related to measures of executive functioning, namely cognitive control, working memory and verbal fluency (Broadway et al., 2019; Fynes-Clinton et al., 2019; Williams et al., 2021). Semantic strategies positively and significantly correlate with better performance in recall tests (Sohlberg and Mateer, 2001; Kirchhoff, 2009) and both short and long-term recall and recognition (Del Valle and Urquijo, 2015). Therefore, they are related to superior verbal memory performance. On the other hand, the serial grouping strategy is considered a passive strategy that depends on auditory and attentional knowledge and has been related to executive functioning to a lesser extent (Broadway et al., 2019; Williams et al., 2021). In addition, together with measures of executive functioning, semantic clustering predicts overall memory performance. For this reason, we consider it important to determine how the ability to use grouping strategies, especially of a semantic type, can determine performance in remembering a list of words. To the best of our knowledge, no studies have been reported on this relationship in subjects BD.

BD subjects present executive monitoring difficulties, closely linked to memory difficulties (Lannoy et al., 2017a, 2018). Such difficulties lead to lower accuracy, as a result of retrieval intrusions and perseveration errors that reduce memory performance (Scaife and Duka, 2009; Sanhueza et al., 2011; Lundervold et al., 2019). Due to the close relationship between verbal memory and executive functioning, the aim of this paper is to find out to what extent executive components, such as the use of strategies in mnemonic tasks (Carbia et al., 2018; Gierski et al., 2020), determine performance in a verbal memory test and how excessive alcohol consumption may affect this performance. We hypothesize that executive ability, assessed by strategies and accuracy errors committed, will determine the performance in verbal memory tests; moreover, we suggest that binge drinking will affect this performance and will relate to poorer executive ability, i.e., less use of strategies and more memory accuracy errors.

## 2. Materials and methods

### 2.1. Participants

One hundred and sixty five first-year university students (51.55% female and 48.45% male) with a mean age of 18.12 ± 0.32 years participated. They were selected according to a non-probabilistic sampling from the university campus and collectively and anonymously completed a questionnaire on sociodemographic information, psychopathological symptomatology and information on the use of other legal and illegal drugs, as well as prescription and non-prescription drugs, was collected in the collectively administered questionnaires that included the Symptom Checklist-90-R (SCL-90R; Derogatis, 1983), the Spanish version of the Alcohol Use Disorders Identification Test (AUDIT; Babor et al., 2001) and the Intensive Drinking Evaluation Instrument (IECI; Cortés et al., 2012). Participants reported via a 10 point Likert scale if they were “in agreement” (10) or “in disagreement” (0) with 16 different statements about the effects of carrying out an alcohol consumption. This information was used to decide on inclusion/exclusion in the study. And it was corroborated during the interview prior to the neuropsychological evaluation. Taking into account the amount of alcohol consumed, body mass index, gender and duration of the drinking episode, the highest blood alcohol concentration (BAC) that a subject would have reached in one of these episodes was estimated (Widmark, 1922; Fitzgerald, 1995).

Each participant was interviewed prior to the neuropsychological assessment. In the interview, information about their pattern of alcohol consumption was verified and the following exclusion criteria were applied: family history of alcoholism (first-and second-degree history) or major psychopathological disorder, personal history of neurological disorders or systemic diseases affecting neurocognitive functioning, regular use of drugs or prescription drugs with psychoactive effects, and motor or sensory impairment that prevented them from taking the tests. Then, they participated in two neuropsychological assessment sessions of 50-min sessions each on separate dates. This study is part of a larger project that used a comprehensive neuropsychological assessment protocol. In this paper we only present data referring to learning/memory tasks.

### 2.2. Instruments

The following instruments were applied:

Symptom Checklist-90-Revised (SCL-90-R; Derogatis, 1983): A self-administered questionnaire composed of a list of 90 items grouped in dimensions such as: depression, anxiety, somatizations, obsessions and compulsions, interpersonal sensitivity, hostility, phobic anxiety, paranoid ideation and psychoticism. In addition, it provides a global index of psychological distress and a global severity index (GSI).

Alcohol Use Disorders Identification Test (AUDIT): Adapted for the Spanish population by Babor et al. (2001). AUDIT was developed by the WHO as a simple screening method of excessive alcohol consumption and it is the most widely used instrument for this purpose. It has 10 questions that enquires about alcohol intake (3 questions about frequency, quantity and binge drinking), potential dependence on alcohol (3 questions), and experience of alcohol-related harm (4 questions).

Intensive Drinking Evaluation Instrument (IECI): Self-registration of consumption in which they report the number of times they drank alcohol during the last 6 months and indicate the number of drinks consumed each day of a week of habitual consumption during those 6 months. They also record the type of alcohol consumed and the time at which each intake was made. This scale presents a good fit in all the studies carried out, ranging from Cronbach’s alpha between 0.900 and 0.913 (Cortés et al., 2012; Motos, 2013).

For memory evaluation, the following instruments were applied:

Test de Aprendizaje Verbal España-Complutense (TAVEC) (Benedet and Alejandre, 2014): This test is equivalent to the California Verbal Learning Test-CVLT (Delis et al., 1987). It is applied to assess learning stability, short and long-term memory, retention, susceptibility to impaired learning interference and recognition. It provides a measurement of the learning strategies employed (serial and semantic). It consists of three-word lists: a learning list (List A), which is presented over five consecutive trials; an interference list (List B), which is presented once after the five previous trials; and a recognition list. Lists A and B are based on the learning of a list of 16 words grouped into four different semantic categories, with List B sharing two categories from the initial list and adding two new categories. The recognition list consists of 44 words. Free and cued recall of list A are tested immediately (short-delay recall), and again after 20 min (long-delay recall). In cued recall trials, the examiner prompts the subjects with the word category.

Logical Memory (WMS-III) (Wechsler, 1997): It is a two-part test in which two short stories (A and B) are read to the subject, with a second presentation of story B. After the presentation of each story, the subject is asked to try to reproduce the story as accurately as possible, emphasizing the importance of all the words in the text read (Logical Memory I). After 30 min, they must repeat what they remember from both stories (Logical memory II) and are given a recognition trial (15 questions per story in which they must say whether the information provided is true or false). Immediate and delayed verbal memory, functions related to hippocampal functioning are therefore assessed (De Toledo-Morrell et al., 2000; Papanicolaou et al., 2002; Wong et al., 2021).

Variables in this research are classified into two groups (Supplementary Table S1). Variables explicitly measuring memory performance in recall and recognition trials are in the first group. Variables which can be considered as indicators of executive skills, as is the case of those measuring strategies or accuracy errors, are in the second group.

### 2.3. Statistical analysis

Pearson’s correlation was calculated to analyze the relationship between participants’ BAC and verbal memory performance. Subsequently, a cluster analysis was conducted to describe and classify participants into groups sharing similar performance profiles in verbal memory and BAC levels achieved. This cluster analysis combines factorial methods and grouping techniques (Lebart et al., 1995) in four phases of analysis: in the first, a Principal Component Analysis (PCA) is carried out, this facilitates the identification of components (dimensions) underlying the data (Lebart et al., 1995; Pardo and Del Campo, 2007; Husson et al., 2010a,b); in the second phase, an agglomerative hierarchical classification is performed using Ward’s method (Pardo and Del Campo, 2007). Since the variables used to define the factorial plane are quantitative, the Euclidean distance between all the elements to be classified (160 participants) is determined using the first factorial plane obtained through the PCA. The third phase is a classification through mobile centers using the K-means method where, in addition, a validation of the identified groups is determined through hypothesis testing. Finally, the fourth phase includes the description of each of the groups obtained from the previously established continuous and categorical variables. If the variables are continuous, the mean of each group is compared with the general mean; in the case of categorical variables, the percentage of each group is compared with the general percentage. The v.test statistic (Husson et al., 2010a) is used to determine whether they are statistically significant. Data analysis was conducted with R version 3.2.4 (R Core Team, 2020) and the FactoMineR package (Lê et al., 2008) version 1.32 (Husson et al., 2016) was used for PCA and Cluster Analysis.

Data analysis was conducted using R version 3.2.4 (R Core Team, 2020). For PAC and Cluster Analysis, FactoMineR (Lê et al., 2008) version 1.32 (Husson et al., 2016) was used.

## 3. Results

Pearson’s correlation analysis demonstrates correlations between the use of memory strategies and performance in these tests (Table 1). Thus, for example, to the extent that participants were more skilled at using semantic strategies, their performance in the memory tests was better; on the contrary, the commission of perseverative errors or the inefficiency of monitoring processes was related to a worse performance in the memory tests. Finally, Table 1 also shows how the BAC achieved correlates negatively with both memory performance and total strategy use.

**Table 1:** Statistically significant correlations between BAC, MP, and ECMP (*p* < 0.05).

	BAC	TAVEC_ERRORS	TAVEC_SD_FR_SMC	TAVEC_IR_A_SMC	TAVEC_IR_B_SMC	TAVEC_LD_FR_SMC	TAVEC_IR_B_SRC	TAVEC_RFP	TAVEC_CRI	TAVEC_FRI	TAVEC_P	TAVEC_TOTSMC	TAVEC_TOTCS
BAC													−0.21
TAVEC_DISCRIM	−0.22	−0.29	0.32	0.35		0.36		−0.72	−0.24			0.37	0.38
TAVEC_SDCR	−0.22	−0.34	0.62	0.59	0.21	0.63	−0.21	−0.29	−0.26	−0.18	−0.22	0.65	0.66
TAVEC_LDCR	−0.17	−0.34	0.52	0.53	0.19	0.57		−0.42	−0.25	−0.20	−0.16	0.58	0.65
TAVEC_RS	−0.29	−0.21	0.35	0.36		0.35		−0.20	−0.20			0.38	0.42
TAVEC_RS_LDCR		−0.29	0.43	0.43	0.16	0.49		−0.40	−0.20	−0.18		0.48	0.54
TAVEC_RS_LDFR		−0.35	0.46	0.44	0.19	0.57		−0.27	−0.23	−0.26	−0.18	0.51	0.59
TAVEC_IR1	−0.20	−0.17	0.28	0.52	0.29	0.29						0.48	0.57
TAVEC_FTA5	−0.29	−0.29	0.38	0.48	0.16	0.36		−0.27	−0.26	−0.18		0.47	0.61
TAVEC_TFRA5	−0.28	−0.26	0.36	0.60	0.31	0.32		−0.19	−0.21	−0.16		0.55	0.74
TAVEC_IRB					0.27								0.18
TAVEC_LB_IR1A		−0.21	0.17	0.28		0.20						0.25	0.26
TAVEC_SDFR	−0.21	−0.34	0.66	0.58	0.22	0.50		−0.34	−0.27	−0.20	−0.19	0.63	0.70
TAVEC_SDFR_FTA5			−0.42	−0.25		−0.27	0.19	0.17				−0.31	−0.25
TAVEC_LDFR	−0.24	−0.39	0.54	0.53	0.21	0.64		−0.31	−0.28	−0.28	−0.20	0.60	0.68
LM_LC					−0.22					0.19			−0.18
LMI_FR1	−0.22	−0.16			0.22				−0.20				0.29
LMI_TRS3	−0.21	−0.21	0.16		0.18				−0.23				0.33
LMI_TST	−0.21				0.21				−0.17				0.20
LMII_RP													
LMII_TRS_AB		−0.19			0.19				−0.22				0.26
LMII_TST													

Cluster analysis was used to identify the possible groups into which the 160 participants fall. Based on the agglomerative hierarchical classification, three groupings were identified: cluster 1 is made up of 42 people (26.25%), cluster 2 of 69 people (43.12%), and cluster 3 of 49 people (30.63%).

Significant differences between three groups were estimated by variance analysis for the 42 previously defined variables. Table 2 shows the variables, including BAC. Differences between groups manifest an effect size greater than 0.8 which allows us to affirm that these variables behave differently depending on the group. In other words, the configuration of the groups estimated through the cluster analysis can be considered valid.

**Table 2:** Three estimated groups ANOVA for RM, CERM, and BAC.

Variable	*F* ^a^	Prob	*R*^2^	*d* (IC 0.95)
TAVEC_LDFR	207.53	0.000	0.72	3.24 (2.65; 3.84)
TAVEC_LDCR	198.20	0.000	0.71	3.17 (2.58; 3.75)
TAVEC_SDCR	146.99	0.000	0.65	2.73 (2.20; 3.26)
TAVEC_SDFR	140.53	0.000	0.64	2.67 (2.15; 3.19)
TAVEC_TOTCS	89.81	0.000	0.53	2.13 (1.68; 2.59)
TAVEC_RS_LDFR	77.50	0.000	0.49	1.98 (1.54; 2.42)
TAVEC_RS_LDCR	67.20	0.000	0.45	1.84 (1.42; 2.27)
TAVEC_TOTSMC	64.48	0.000	0.44	1.81 (1.39; 2.23)
TAVEC_TFRA5	59.57	0.000	0.42	1.74 (1.32; 2.15)
TAVEC_IR_A_SMC	49.78	0.000	0.38	1.59 (1.19; 1.99)
TAVEC_RS	49.50	0.000	0.38	1.58 (1.18; 1.98)
TAVEC_LD_FR_SMC	47.89	0.000	0.37	1.56 (1.16; 1.95)
TAVEC_DISCRIM	46.91	0.000	0.36	1.54 (1.15; 1.94)
TAVEC_SD_FR_SMC	45.30	0.000	0.36	1.51 (1.12; 1.91)
TAVEC_FTA5	37.49	0.000	0.31	1.38 (1.00; 1.76)
LMI_TRS3	23.27	0.000	0.22	1.09 (0.73; 1.44)
LMI_FR1	19.44	0.000	0.19	0.99 (0.64; 1.34)
LMII_TRS_AB	19.42	0.000	0.19	0.99 (0.64; 1.34)
TAVEC_IR1	19.04	0.000	0.18	0.98 (0.63; 1.33)
TAVEC_SDFR_FTA5	17.40	0.000	0.17	0.94 (0.59; 1.28)
LMII_TST	14.48	0.000	0.14	0.86 (0.52; 1.20)
TAVEC_ERRORS	13.86	0.000	0.14	0.84 (0.50; 1.18)
LMI_TST	13.49	0.000	0.14	0.83 (0.49; 1.16)
TAVEC_CRI	8.92	0.000	0.09	0.67 (0.34; 1.00)
TAVEC_RFP	7.60	0.001	0.08	0.62 (0.29; 0.95)
TAVEC_IR_B_SMC	6.27	0.002	0.06	0.56 (0.24; 0.89)
TAVEC_P	5.50	0.005	0.05	0.53 (0.20; 0.85)
TAVEC_LB_IR1A	5.38	0.005	0.05	0.52 (0.20; 0.84)
LM_LC	5.27	0.006	0.05	0.52 (0.19; 0.84)
BAC	5.24	0.006	0.05	0.51 (0.19; 0.84)
TAVEC_IR_B_SRC	4.78	0.009	0.05	0.49 (0.17; 0.81)
LMII_RP	4.39	0.013	0.04	0.47 (0.15; 0.79)
TAVEC_FRI	3.94	0.020	0.04	0.45 (0.13; 0.77)
TAVEC_IRB	3.54	0.030	0.03	0.42 (0.10; 0.74)
LMII_TRS	2.52	0.081	0.02	
TAVEC_IR_A_SRC	1.38	0.251	0.00	
TAVEC_LDCR_LDFR	0.77	0.462	0.00	
TAVEC_TOTSRC	0.76	0.465	0.00	
TAVEC_LDFR_SDFR	0.47	0.620	−0.01	
TAVEC_SD_FR_SRC	0.39	0.673	−0.01	
TAVEC_LD_FR_SRC	0.25	0.779	−0.01	
TAVEC_SDCR_LDCR	0.10	0.907	−0.01	

a gl_1_ = 2 y gl_2_ = 158.

Finally, Supplementary Table S2 lists the variables that characterize each of the three groups obtained.

If we take the BAC variable as a reference, whose overall mean is 0.08, we can observe that group 1 is characterized by a value of this variable of 0.12; while group 3 is characterized by a BAC value of 0.06. This means that group 1 includes those with binge drinking, while group 3 includes those who do not report binge drinking episodes. The BAC variable does not characterize group 2 in any way.

Group 1, which is characterized by a high BAC, it is also defined by negative values in variables measuring verbal memory performance (MP). In contrast, group 3, defined by a lower BAC, includes positive values in memory task performance.

In the case of variables related to executive components of memory performance (ECMP), such as use of strategies or commission errors, the values they acquire depending on whether they are in group 1 or 3 are different. For example, group 1 includes participants who made many errors (TAVEC_ERRORS), especially false positives and intrusions in cued recall (TAVEC_RFP; TAVEC_CRI) and are not very skilled at using memory strategies, especially semantic ones (TAVEC_TOTCS; TAVEC_TOTSMC). In contrast, group 3 includes participants who use these memory strategies efficiently by applying efficient memory strategies and make fewer mnemonic errors.

Moreover, 55.6% of the variables characterizing group 1 demonstrate a size effect greater than 0.80, which means they are highly relevant. Thus, Group 1 is made up of participants who consume alcohol intensively, whose performance in memory tests are lower than average and who make more accuracy errors. Group 3 is a specular clustering with that observed in Group 1. That is, subjects characterized by lower BAC values who perform well in the memory tests and make few errors in them. Likewise, the variables characterizing this group are very relevant, since the effect size observed is mostly higher than 0.8.

Table 3 shows the individual scores of the most characteristic subjects from the estimated clusters. Participant 73 represents cluster 3, with no alcohol consumption (BAC = 0.00). Their scores in the memory components (MP) and use of predominantly semantic strategies (CEM) are higher, and they do not score in memory error commission. On the other hand, subject 153, from cluster 1, with a BD consumption pattern (BAC = 0.18), exhibits the lowest scores in the strategy use variables (ECMP), a high commission of errors, and lower scores in the memory variables (MP).

**Table 3:** Most characteristic subjects of estimated clusters.

Classification	Variables	Cluster 1 (Subject 153)	Cluster 3 (Subject 73)
	BAC	0.18	0.00
MP	TAVEC_IR1	5.00	7.00
	TAVEC_IRB	5.00	5.00
	TAVEC_SDFR	11.00	16.00
	TAVEC_SDCR	12.00	15.00
	TAVEC_LDFR	11.00	16.00
	TAVEC_LDCR	12.00	16.00
	TAVEC_DISCRIM	95.45	100.00
	TAVEC_LB_IR1A	0.00	40.00
	LMI_FR1	26.00	38.00
	LMI_TST	12.00	18.00
	LMII_TST	9.00	12.00
	LMII_RP	96.67	92.50
	TAVEC_LDFR_SDFR	0.00	0.00
	LM_LC	4.00	2.00
	TAVEC_TFRA5	50.00	61.00
	TAVEC_RS	14.00	16.00
	TAVEC_RECONAC_RLLP	−21.42	0.00
	TAVEC_RS_LDCR	−14.29	0.00
	TAVEC_RCLLP MENOS RLLP	−8.33	0.00
	LMI_TRS3	40.00	59.00
	LMII_TRS_AB	29.00	37.00
	LMII_TRS	26.00	27.00
	TAVEC_FTA5	13.00	16.00
	TAVEC_SDCR_LDCR	0.00	6.67
	TAVEC_SDFR_FTA5	18.18	0.00
ECMP	TAVEC_P	5.00	0.00
	TAVEC_FRI	0.00	0.00
	TAVEC_CRI	1.00	0.00
	TAVEC_RFP	0.00	0.00
	TAVEC_ERRORS	6.00	0.00
	TAVEC_TOTSRC	8.00	10.00
	TAVEC_TOTSMC	13.00	45.00
	TAVEC_TOTCS	21.00	55.00
	TAVEC_IR_A_SRC	6.00	7.00
	TAVEC_IR_B_SRC	1.00	2.00
	TAVEC_IR_A_SMC	7.00	24.00
	TAVEC_IR_B_SMC	0.00	0.00
	TAVEC_SD_FR_SMC	2.00	10.00
	TAVEC_LD_FR_SMC	4.00	11.00
	TAVEC_SD_FR_SRC	1.00	0.00
	TAVEC_LD_FR_SRC	0.00	1.00

## 4. Discussion

The aim of our study was to find out to what extent an indicator of executive functioning, such as the accurate use of strategies in the memory process (Carbia et al., 2018; Gierski et al., 2020), might determine the performance of the participants in a verbal memory test and how excessive alcohol consumption can affect this performance. The results of this study indicate that there is a relationship between the executive components of memory performance and global memory performance. In addition, the participants are grouped into two significantly different clusters according to their drinking patterns, which represent specular images regarding TAVEC performance.

The participants who consume alcohol according to a BD pattern are characterized by poorer global performance in memory and ECMPs. They use less semantic grouping strategies, make more errors, more intrusions, and more false positives. They are characterized by using serial grouping strategies, which are less efficient. In contrast, the participants characterized by not binge drinking show good performance on memory tests and on ECMP-related indices. They use semantic grouping strategies. In other words, they engage cognitive abilities to organize and classify words based on their semantic similarities. These strategies allow for the identification and grouping of words into categories based on their related meanings. In addition, cognitive control is applied to avoid perseveration, intrusion, or false positives errors. Furthermore, they typically do not employ serial clustering strategies. Data collected in our research regarding the set of strategies and errors made in the memory tests allow us to configure different characterizations of the groups according to their pattern of alcohol consumption. As we have already noted, it seems the use of semantic strategies requires more cognitive effort than serial grouping strategies (Introzzi et al., 2010) and, moreover is regarded as one of the most efficient resources in memory tests (Sohlberg and Mateer, 2001). In our study, we observed that the participants in the BD cluster used less semantic strategies than non-drinkers or low-consumption-drinkers, like that observed by Squeglia et al. (2009), who found higher semantic strategy scores during the first learning stages in those participants who do not drink alcohol. Our results show that the use of serial strategies characterize BD subjects more than non-BD subjects, something that could be explained by the latter’s use of semantic strategies which, as we have pointed out, require better executive skills and are therefore more decisive in determining the outcome in word list learning results.

The importance of the engagement of prefrontal regions in strategy implementation has been described using the California Verbal Learning Test (CVLT) (Baldo et al., 2002; Alexander et al., 2003). The results of our study provide neuropsychological evidence that the overall use of grouping strategies (TAVEC_TOTCS) is lower or less frequent, as is the detriment in semantic grouping (TAVEC_TOTSMC), in BD subjects (Winward et al., 2014). In fact, there is evidence that BD affects prefrontal regions and thus executive functioning (López-Caneda et al., 2014; Lees et al., 2019).

In addition, our results show that participants in the BD group commit a higher number of intrusions, perseverations, and false positives. It is interesting to note that the performance of BD subjects in word list learning tests is characterized by displaying perseveration errors (Schweinsburg et al., 2010; Sanhueza et al., 2011; Parada et al., 2012; Gierski et al., 2020) and intrusions (Lundervold et al., 2019; Gierski et al., 2020). In addition, studies using this same test have also found that BD subjects make these same types of errors more frequently than non-drinkers (García-Moreno et al., 2008, 2009). These data could suggest that BD subjects may have difficulties in monitoring the test, i.e., in knowing precisely what information they have or have not been given previously. The ability to update and contrast presented information with that already stored depends on working memory (Carbia et al., 2017). The impairment of monitoring processes in BD has already been documented in electrophysiological and behavioral studies of executive function (Lannoy et al., 2017a,b; Lannoy et al., 2018), linking memory difficulties, at least in part, to executive processes.

The data from our study describe decrements in ECMP and overall impaired memory performance in BD participants. In fact, the long-term effects of BD in young people are associated with decreased learning ability and negative effects on verbal memory (Carbia et al., 2017; Lannoy et al., 2019; Lees et al., 2019). In line with the findings of our study, BD participants present impaired performance in verbal memory ability compared to non-drinkers (Winward et al., 2014). Various neuropsychological studies have described the relationship between BD in adolescents and verbal memory performance. In particular, worse performance is found in learning and verbal and visuospatial memory tests, reflecting verbal and non-verbal information retrieval deficits (Brown et al., 2000). Other studies, such as those by Mota et al. (2013) and Parada et al. (2011), observed differences in memory performance in both short and long-term recall in Wechsler Memory Scale (WMS) subtests, with lower performance in BD adolescents. However, these studies found no significant differences on the RAVLT (Rey Auditory Verbal Learning Test), a word list learning test with a very similar structure to the TAVEC test but using semantically unrelated words. Other studies, which used unrelated word lists, also found no differences in recall (Hartley et al., 2004; Carbia et al., 2017), which may mean that, as it does not require an executive skill such as the use of semantic strategies, differences in performance are not significant (Hartley et al., 2004; Carbia et al., 2017).

However, studies using semantically related word tests found young BD adults perform less well compared to the control group (García-Moreno et al., 2008, 2009; Sneider et al., 2013; Winward et al., 2014). Lower performance on story memory and semantically related word list learning tests has also been verified in longitudinal studies in young people who maintained a stable pattern of intensive alcohol consumption from adolescence to early adulthood (Hanson et al., 2011; Carbia et al., 2017). In the study by Gierski et al. (2020), free recall and immediate cued recall scores are markedly lower among BD subjects, suggesting difficulty in the process of retrieving verbal information from episodic memory. Overall memory scores were significantly lower for the BD group. Using visual material, in a verbal memory test, free and paired recall associated with concrete and abstract stimuli was assessed. Of these, BD youth demonstrated lower performance in the tests of learning pairs associated with abstract figures (Scaife and Duka, 2009) and in the recall of common drawn objects (Hartley et al., 2004).

This work is not without limitations. The main one is that although an effort has been made to measure alcohol consumption as reliably as possible, the calculated BAC is an estimate based on self-reports of consumption. In addition, young university students do not represent the entire population of young people who practice heavy alcohol consumption. Their higher academic level implies a certain type of cognitive training that can be reflected in their performance on memory tests. Therefore, we cannot generalize the results. This effect could be different, perhaps even greater, among young people with a lower academic level.

To conclude, our results demonstrate that there is a clear correlation between executive abilities displayed during the verbal memory process and the performance of these tests. Non-BD participants capable of using semantic grouping strategies or efficiently supervising the process to avoid perseverations or false positives, obtain better punctuations at variables measuring verbal memory performance. In contrast, intensive alcohol consumption its associated with poorer executive function. According to our results, BD participants display fewer executive abilities, such as semantic grouping strategies or error commission and as a result they have worse verbal memory performance than non-BD participants.

## Data availability statement

The raw data supporting the conclusions of this article will be made available by the authors, without undue reservation.

## Ethics statement

The current research project was approved by the Clinical Research Ethics Committee of San Carlos Clinical Hospital in Madrid (Ref. 19/121-E_BC). The studies were conducted in accordance with local regulations and institutional requirements. Participants provided written informed consent to participate in this study.

## Author contributions

FC, LG, and MC: designed the study. CP and PG: collected the data. CP and XO: analyzed and interpreted the data. CP, LG, and MC: wrote the manuscript. All authors contributed to the article and approved the submitted version.
