# Supplementary material for: Verbal memory and executive components of recall in adolescent binge drinkers
Source: Front Psychol. 2023 Oct 23;14:1239716. doi: 10.3389/fpsyg.2023.1239716 (PMC10626472; doi:10.3389/fpsyg.2023.1239716)
Supplement: Supplementary file 1 [file Table_1.DOCX]

*Supplementary Material*

Verbal Memory and executive components of recall in adolescent binge drinkers

Claudia Porras Truque^1*^, Luis Miguel García Moreno^1*^, Patricia Mateos Gordo^1^, Xavier Ordoñez Camacho^1^, Fernando Cadaveira^2^ y Montserrat Corral ^2^

^1^Department of Psychobiology and Methodology in Behavioral Sciences, Universidad Complutense de Madrid (UCM), Spain.

^2^Department of Clinical Psychology and Psychobiology, Universidad de Santiago de Compostela (USC), Santiago de Compostela, Spain

*** Correspondence:** [clporras@ucm.es](mailto:clporras@ucm.es)

# 2. Supplementary Tables

Supplementary Table 1. Memory and executive performance variables in recall and recognition trials

| **Classification** | **Variable** | **Label** |
| --- | --- | --- |
|  | Blood Alcohol Concentration | BAC |
| Memory Performance (MP) | TAVEC: Immediate recall: First learning trial | TAVEC_IR1 |
|  | TAVEC: Immediate recall: Interference list B | TAVEC_IRB |
|  | TAVEC: Short delay: Free recall | TAVEC_SDFR |
|  | TAVEC: Short delay: Cued recall | TAVEC_SDCR |
|  | TAVEC: Long delay: Free recall | TAVEC_LDFR |
|  | TAVEC: Long delay: Cued recall | TAVEC_LDCR |
|  | TAVEC: Discrimination index | TAVEC_DISCRIM |
|  | TAVEC: comparison between list B recall and first learning trial immediate recall from list A | TAVEC_LB_IR1A |
|  | Logical Memory I: First recall total score (the addition of story A and story B first recall) | LMI_FR1 |
|  | Logical Memory I: Total score theme | LMI_TST |
|  | Logical Memory II: Total score theme | LMII_TST |
|  | Logical Memory II: Retention percentage | LMII_RP |
|  | TAVEC: comparison between long delay free recall and short delay free recall | TAVEC_LDFR_SDFR |
|  | Logical Memory: Learning curve | LM_LC |
|  | TAVEC: Total free recall over the five trials of list A | TAVEC_TFRA5 |
|  | TAVEC: recognition score (total correct answers) | TAVEC_RS |
|  | TAVEC: comparison between recognition score and long delay free recall | TAVEC_RS_LDFR |
|  | TAVEC: comparison between recognition score and long delay cued recall | TAVEC_RS_LDCR |
|  | TAVEC: comparison between long delay cued recall and long delay free recall | TAVEC_LDCR_LDFR |
|  | Logical Memory I: Total recall score (the addition of the three stories) | LMI_TRS3 |
|  | Logical Memory II: Total recall score (the addition of A and B units) | LMII_TRS_AB |
|  | Logical Memory II: Total recognition score | LMII_TRS |
|  | TAVEC: Fifth trial list A immediate recall | TAVEC_FTA5 |
|  | TAVEC: Comparison between short delay cued recall and long delay cued recall | TAVEC_SDCR_LDCR |
|  | TAVEC: Comparison between short delay free recall and fifth trial list A immediate recall | TAVEC_SDFR_FTA5 |
| Executive Components of Memory Performance (ECMP) | TAVEC: Perseverations | TAVEC_P |
|  | TAVEC: Free recall intrusions | TAVEC_FRI |
|  | TAVEC: Cued recall intrusions | TAVEC_CRI |
|  | TAVEC: Recognition false positives | TAVEC_RFP |
|  | TAVEC: Total errors | TAVEC_ERRORS |
|  | TAVEC: Total serial clustering | TAVEC_TOTSRC |
|  | TAVEC: Total semantic clustering | TAVEC_TOTSMC |
|  | TAVEC: Total clustering strategies | TAVEC_TOTCS |
|  | TAVEC: immediate recall list A serial clustering | TAVEC_IR_A_SRC |
|  | TAVEC: immediate recall list B serial clustering | TAVEC_IR_B_SRC |
|  | TAVEC: immediate recall list A semantic clustering | TAVEC_IR_A_SMC |
|  | TAVEC: immediate recall list B semantic clustering | TAVEC_IR_B_SMC |
|  | TAVEC: short delay free recall semantic clustering | TAVEC_SD_FR_SMC |
|  | TAVEC: long delay free recall semantic clustering | TAVEC_LD_FR_SMC |
|  | TAVEC: short delay free recall serial clustering | TAVEC_SD_FR_SRC |
|  | TAVEC: long delay free recall serial clustering | TAVEC_LD_FR_SRC |

Supplementary Table 2. Characterization variables for estimated clusters.

| **Classification** | **Variables** | **Mean (sd) Overall** | **Cluster 1 (n=42)** | | | | **Cluster 2 (n=69)** | | | | **Cluster 3 (n=49)** | | | |
| --- | --- | --- | --- | --- | --- | --- | --- | --- | --- | --- | --- | --- | --- | --- |
|  |  |  | **Mean (sd)** | **v.test** | **p.value** | **d (IC .95)** | **Mean (sd)** | **v.test** | **p.value** | **d (IC .95)** | **Mean (sd)** | **v.test** | **p.value** | **d (IC .95)** |
|  | BAC | 0.08 (0.09) | 0.12 (0.11) | 2.91 | 0.004 | 0.47 (0.15; 0.79) |  |  |  |  | 0.06 (0.07) | -2.26 | 0.024 | 0.36 (0.04; 0.68) |
| MP | LM_LC | 5.25 (2.71) |  |  |  |  |  |  |  |  | 4.22 (2.33) | -3.16 | 0.002 | 0.51 (0.19; 0.84) |
|  | LMI_TST | 15.56 (2.85) | 14.76 (3.07) | -2.1 | 0.036 | 0.34 (0.02; 0.65) | 14.89 (2.64) | -2.62 | 0.009 | 0.42 (0.10; 0.74) | 17.20 (2.19) | 4.83 | 0.000 | 0.82 (0.48; 1.16) |
|  | LMII_RP | 89.90 (9.09) |  |  |  |  |  |  |  |  | 93.01 (5.81) | 2.87 | 0.004 | 0.46 (0.1 4; 0.78) |
|  | LMII_TST | 10.02 (2.26) |  |  |  |  | 9.43 (2.26) | -2.93 | 0.003 | 0.47 (0.15; 0.80) | 11.37 (1.49) | 4.98 | 0.000 | 0.85 (0.51; 1.19) |
|  | TAVEC_RS | 15.33 (0.97) | 14.33 (1.11) | -7.71 | 0.000 | 1.53 (1.14; 1.92) | 15.57 (0.67) | 2.77 | 0.006 | 0.45 (0.13; 0.77) | 15.84 (0.47) | 4.38 | 0.000 | 0.73 (0.40; 1.07) |
|  | TAVEC_RS_LDCR | -10.25 (9.71) | -19.86 (10.79) | -7.43 | 0.000 | 1.45 (1.06; 1.83) |  |  |  |  | -2.30 (3.96) | 6.85 | 0.000 | 1.28 (0.91; 1.65) |
|  | TAVEC_RS_LDFR | -11.57 (10.29) | -21.75 (10.30) | -7.43 | 0.000 | 1.44 (1.06; 1.83) |  |  |  |  | -2.43 (3.78) | 7.43 | 0.000 | 1.44 (1.06; 1.83) |
|  | TAVEC_FTA5 | 14.08 (1.64) | 12.69 (1.54) | -6.38 | 0.000 | 1.16 (0.80; 1.52) |  |  |  |  | 15.16 (1.3) | 5.53 | 0.000 | 0.97 (0.62; 1.32) |
|  | TAVEC_TFRA5 | 58.56 (7.06) | 52.02 (5.20) | -6.96 | 0.000 | 1.31 (0.94; 1.68) |  |  |  |  | 64.37 (6.11) | 6.88 | 0.000 | 1.29 (0.92; 1.66) |
|  | TAVEC_SDFR_FTA5 | 8.09 (13.80) | 16.60 (14.8) | 4.63 | 0.000 | 0.78 (0.45; 1.12) |  |  |  |  | 0.96 (10.11) | -4.32 | 0.000 | 0.72 (0.39; 1.06) |
|  | LMI_TRS3 | 50.45 (8.80) | 46.64 (8.40) | -3.25 | 0.001 | 0.53 (0.21; 0.85) | 48.36 (8.22) | -2.64 | 0.008 | 0.43 (0.11; 0.75) | 56.71 (6.3) | 5.95 | 0.000 | 1.06 (0.71; 1.42) |
|  | LMII_TRS_AB | 32.06 (6.26) | 29.52 (6.20) | -3.04 | 0.002 | 0.49 (0.17; 0.81) | 30.67 (6.14) | -2.45 | 0.014 | 0.39 (0.08; 0.71) | 36.2 (4.09) | 5.54 | 0.000 | 0.97 (0.62; 1.32) |
|  | TAVEC_DISCRIM | 97.57 (3.11) | 94.43 (3.62) | -7.59 | 0.000 | 1.49 (1.10; 1.88) | 98.34 (2.07) | 2.75 | 0.006 | 0.44 (0.12; 0.76) | 99.17 (1.64) | 4.28 | 0.000 | 0.72 (0.38; 1.05) |
|  | TAVEC_SDCR | 13.55 (1.86) | 11.26 (1.42) | -9.22 | 0.000 | 2.12 (1.66; 2.57) |  |  |  |  | 15.24 (0.82) | 7.63 | 0.000 | 1.51 (1.12; 1.90) |
|  | TAVEC_LDCR | 13.77 (1.80) | 11.43 (1.33) | -9.75 | 0.000 | 2.40 (1.91; 2.89) |  |  |  |  | 15.47 (0.7) | 7.88 | 0.000 | 1.58 (1.19; 1.98) |
|  | TAVEC_IR1 | 7.43 (1.72) | 6.45 (1.43) | -4.3 | 0.000 | 0.72 (0.39; 1.05) |  |  |  |  | 8.45 (1.82) | 4.95 | 0.000 | 0.85 (0.51; 1.19) |
|  | TAVEC_IRB | 6.39 (1.61) |  |  |  |  | 6.07 (1.47) | -2.16 | 0.030 | 0.35 (0.03; 0.66) | 6.86 (1.78) | 2.46 | 0.014 | 0.39 (0.08; 0.71) |
|  | TAVEC_LB_IR1A | 22.93 (40.74) | 5.66 (29.11) | -3.19 | 0.001 | 0.52 (0.20; 0.84) |  |  |  |  |  |  |  |  |
|  | TAVEC_SDFR | 13.19 (1.93) | 10.95 (1.17) | -8.68 | 0.000 | 1.88 (1.45; 2.30) |  |  |  |  | 15.08 (1.05) | 8.2 | 0.000 | 1.69 (1.28; 2.10) |
|  | TAVEC_LDFR | 13.57 (1.89) | 11.17 (1.31) | -9.56 | 0.000 | 2.29 (1.82; 2.77) |  |  |  |  | 15.45 (0.7) | 8.31 | 0.000 | 1.73 (1.32; 2.15) |
|  | LMI_FR1 | 30.12 (6.54) | 27.57 (6.16) | -2.93 | 0.003 | 0.48 (0.15; 0.80) | 28.61 (6.22) | -2.56 | 0.010 | 0.41 (0.09; 0.73) | 34.47 (4.98) | 5.56 | 0.000 | 0.98 (0.63; 1.32) |
| ECMP | TAVEC_TOTSMC | 35.40 (16.54) | 20.79 (8.42) | -6.64 | 0.000 | 1.23 (0.86; 1.59) |  |  |  |  | 50.14 (15.37) | 7.46 | 0.000 | 1.45 (1.07; 1.84) |
|  | TAVEC_TOTCS | 44.94 (14.02) | 31.86 (8.51) | -7.01 | 0.000 | 1.33 (0.95; 1.70) |  |  |  |  | 58.8 (11.40) | 8.27 | 0.000 | 1.72 (1.31; 2.13) |
|  | TAVEC_IR_A_SMC | 20.50 (10.68) | 11.69 (5.25) | -6.2 | 0.000 | 1.12 (0.76; 1.48) |  |  |  |  | 29.29 (10.95) | 6.88 | 0.000 | 1.29 (0.92; 1.66) |
|  | TAVEC_IR_B_SMC | 1.35 (1.54) |  |  |  |  |  |  |  |  | 1.98 (1.99) | 3.4 | 0.001 | 0.56 (0.23; 0.88) |
|  | TAVEC_IR_B_SRC | 0.78 (0.99) | 1.10 (1.13) | 2.38 | 0.017 | 0.38 (0.06; 0.70) |  |  |  |  | 0.47 (0.79) | -2.65 | 0.008 | 0.43 (0.11; 0.75) |
|  | TAVEC_SD_FR_SMC | 6.14 (3.31) | 3.60 (2.05) | -5.76 | 0.000 | 1.02 (0.67; 1.37) |  |  |  |  | 8.86 (3.07) | 6.87 | 0.000 | 1.29 (0.92; 1.66) |
|  | TAVEC_LD_FR_SMC | 7.40 (3.37) | 4.50 (2.38) | -6.47 | 0.000 | 1.19 (0.82; 1.55) |  |  |  |  | 10.02 (2.37) | 6.5 | 0.000 | 1.19 (0.83; 1.56) |
|  | TAVEC_ERRORS | 6.76 (4.98) | 8.90 (6.02) | 3.23 | 0.001 | 0.53 (0.20; 0.85) |  |  |  |  | 4.00 (2.72) | -4.64 | 0.000 | 0.79 (0.45; 1.12) |
|  | TAVEC_RFP | 0.40 (0.79) | 0.79 (1.21) | 3.69 | 0.000 | 0.61 (0.28; 0.93) |  |  |  |  | 0.20 (0.49) | -2.05 | 0.041 | 0.33 (0.01; 0.64) |
|  | TAVEC_FRI | 1.93 (2.66) |  |  |  |  |  |  |  |  | 1.1 (1.78) | -2.59 | 0.01 | 0.42 (0.1; 0.74) |
|  | TAVEC_CRI | 0.71 (1.34) | 1.19 (1.74) | 2.67 | 0.008 | 0.43 (0.11; 0.75) |  |  |  |  | 0.10 (0.36) | -3.82 | 0.000 | 0.63 (0.30; 0.96) |
|  | TAVEC_P | 3.73 (2.96) |  |  |  |  |  |  |  |  | 2.59 (2.28) | -3.21 | 0.001 | 0.52 (0.20; 0.85) |
|  | | | | | | | | | | | | | | |
